# Inflorescence development in female cannabis plants is mediated by photoperiod and gibberellin

**DOI:** 10.1093/hr/uhae245

**Published:** 2024-09-03

**Authors:** Hanan Alter, Yael Sade, Archit Sood, Mira Carmeli-Weissberg, Felix Shaya, Rina Kamenetsky-Goldstein, Nirit Bernstein, Ben Spitzer-Rimon

**Affiliations:** Institute of Plant Sciences, Agricultural Research Organization, The Volcani Institute, Rishon LeZion, Israel; The Robert H. Smith Faculty of Agriculture, Food and Environment, The Hebrew University of Jerusalem, Rehovot, Israel; Institute of Soil, Water and Environmental Sciences, Agricultural Research Organization, The Volcani Institute, Rishon LeZion, Israel; Institute of Plant Sciences, Agricultural Research Organization, The Volcani Institute, Rishon LeZion, Israel; Institute of Plant Sciences, Agricultural Research Organization, The Volcani Institute, Rishon LeZion, Israel; Institute of Plant Sciences, Agricultural Research Organization, The Volcani Institute, Rishon LeZion, Israel; Institute of Plant Sciences, Agricultural Research Organization, The Volcani Institute, Rishon LeZion, Israel; Institute of Soil, Water and Environmental Sciences, Agricultural Research Organization, The Volcani Institute, Rishon LeZion, Israel; Institute of Plant Sciences, Agricultural Research Organization, The Volcani Institute, Rishon LeZion, Israel

## Abstract

In cannabis seedlings, the initiation of solitary flowers is photoperiod-independent. However, when cannabis reaches the adult stage, short-day photoperiod (SD) triggers branching of the shoot apex and a reduction in internode length, leading to development of a condensed inflorescence. We demonstrate that SD affects cannabis plants in two distinct phases: the first includes rapid elongation of the internodes and main stem, and occurring from Day 5 to Day 10 of plant cultivation under SD; in the second phase, elongation of newly developed internodes ceases, and a condensed inflorescence is formed. Exposure of plants to alternating photoperiods revealed that inflorescence onset requires at least three consecutive days of SD, and SD is consistently required throughout inflorescence maturation to support its typical condensed architecture. This photoperiod-dependent morphogenesis was associated with a decrease in gibberellin (GA_4_) and auxin levels in the shoot apex. Reverting the plants to a long-day photoperiod (LD) increased GA_4_ and auxin levels, leading to inflorescence disassembly, internode elongation, and subsequent resumption of LD growth patterns. Similar developmental patterns were observed under SD following the application of exogenous GA (and not auxin), which also impeded inflorescence development. Nevertheless, additional studies will help to further evaluate auxin’s role in these developmental changes. We propose a crucial role for GA in sexual reproduction and inflorescence development in female cannabis by mediating photoperiod signaling in the inflorescence tissues.

## Introduction

*Cannabis sativa* L. is cultivated globally for medicinal and industrial purposes, e.g. as a fiber source and for seed-oil production, medicinal uses, and recreational consumption [1–4]. Plant flowering and inflorescence development affect various aspects of cannabis production. For instance, the timing and development of the inflorescence are crucial in hemp cultivation as it directly influences fiber yield and quality, which peak just after flowering [5]. For seed-oil production, the number of seeds derived from inflorescence architecture is a major determinant of yield, whereas in medical or recreational cannabis, the primary product is the inflorescence itself, which accumulates therapeutic compounds such as cannabinoids, terpenes, and flavonoids [1, 6, 7]. Therefore, understanding reproductive development and identifying factors that regulate plant architecture and inflorescence formation will contribute to cannabis production, breeding, and propagation [6, 8–11].

In cannabis propagated from seeds, reproductive development consists of two stages. Following the juvenile-to-adulate transition, solitary axillary flowers are initiated under a long-day photoperiod (LD). This morphogenetic change is governed by internal signals and preceded by dramatic reproduction-related transcriptional changes in flowering-related genes [8, 10, 12]. Later, in adult plants, each of the newly developed phytomers contains a solitary flower regardless of photoperiod length. Once plants are exposed to a short-day photoperiod (SD), the architecture of female cannabis plants changes, while the basic phytomer structure is maintained [8, 10]. Development of the condensed inflorescence is an outcome of major structural changes in the shoot apex, including intense branching, reduced number of leaflets, and shortening of the petioles and nodes, which result in clustering of the solitary flowers [8, 10].

Gibberellins (GAs) regulate numerous aspects of plant reproductive development and their effect can be either promotive, non-existent, or inhibitory [13–15]. One of the most pronounced reproduction-related functions of GAs is flower induction in *Arabidopsis* under non-inductive SD conditions, as was demonstrated in *Arabidopsis* mutant late-flowering phenotypes with reduced active GA levels, or defective GA signaling [16]. In contrast, GAs have opposite effects on flower transition in some plant species, and exogenous-GA application delayed flowering time in tomato, *Fuchsia* and *Pisum sativum* [17–19]. GA is also the primary regulator of bolting—the process of stem elongation before or during flower and inflorescence development, in many plant species. During bolting, GA levels and signaling are stimulated by external signals, such as LD, that lead to elongation of growing internodes via enhanced cell elongation due to relaxation of the cell wall [15]. However, in some rosette species, application of GA promotes cell division in non-lignified tissues below the meristem during bolting [15]. Similarly, in cereals, in addition to cell elongation, GA induces cell division in intercalary meristems, which also leads to internode elongation [20]. In cannabis, GAs are involved in sex determination, and they are infrequently employed for the production of feminized seeds, as they can promote the initiation of male flowers on female plants when administered at the appropriate concentration [21, 22]. Nonetheless, for practical applications, this approach has mostly been replaced by the more efficient ethylene-signaling inhibitor silver thiosulfate [21, 22].

We propose that GA plays a pivotal role in regulating inflorescence development in cannabis. Our study revealed that in comparison to LD, under SD, GA and auxin are accumulated to lower levels in the shoot apex, thereby inducing robust branching and node shortening to produce clustering of solitary flowers and inflorescence development. Conversely, a return to LD prompts an elevation in GA and auxin levels, leading to internode elongation within the inflorescence, and resulting in its disintegration and the reestablishment of LD growth patterns. Notably, a similar response was observed following the exogenous application of GA under SD conditions. These findings underscore the requirement of continuous exposure to SD to sustain inflorescence development, likely by preserving low GA levels in the inflorescence tissues.

## Results

### Effects of photoperiod on plant architecture and inflorescence development

**Figure 1:**
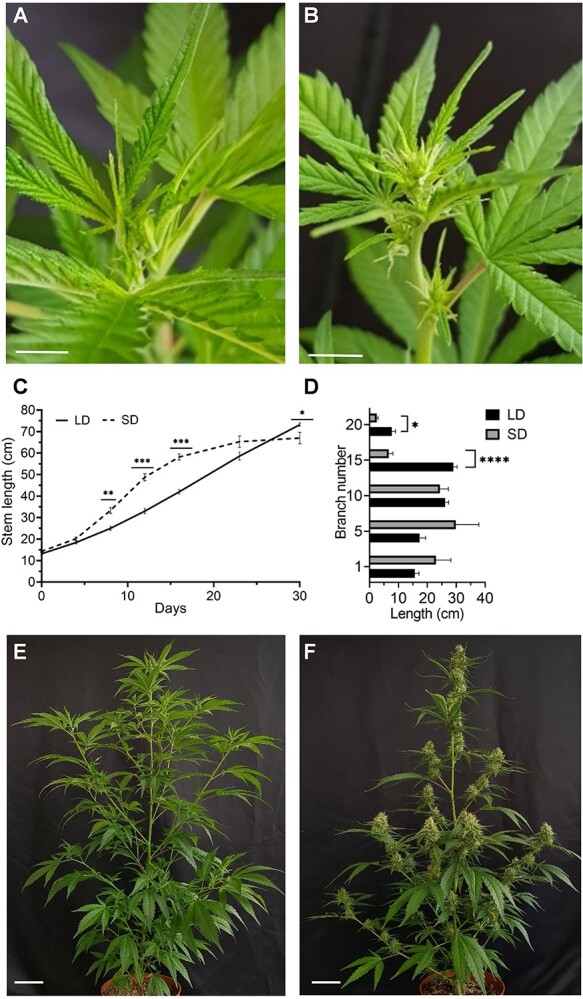
Development of cannabis plants under different photoperiods. Long day (LD – 18 h/6 h, short day (SD – 12 h/12 h. **A,** Plant after 1 week of growth under LD. Bar = 2 cm. **B,** Plant after 1 week of growth under SD. Bar = 2 cm. **C,** Stem elongation during 30 days of plant growth under LD and SD. **D,** Lateral branch length after 30 days of plant growth under LD and SD (measured for branches from node numbers: 1, 5, 10, 15, 20; *n* = 3–5). **E,** Plant after 30 days of growth under LD. Bar = 20 cm. **F,** Plant after 30 days of growth under SD. Bar = 10 cm. Significant differences were assessed by unpaired *t*-test; ^*^^,^  ^**^^,^  ^***^^,^  ^****^P ≤ 0.05, 0.01, 0.001, and 0.0001, respectively. Presented data are averages ± SE.

To evaluate the effects of photoperiod on plant architecture and inflorescence development, we compared responses of plants that were grown under continuous LD photoperiod to plants that were switched to a SD photoperiod for a period of 30 days (Table S1). In plants grown under continuous LD conditions, no inflorescence development was recorded (Fig. 1A), whereas in plants grown under SD photoperiod, inflorescences became visible 7 days following the transition to SD (Fig. 1B). During the first 14 days, plants grown under SD showed significantly accelerated growth compared to plants grown under LD (Fig. 1C). Then, the height of the plants grown under SD stabilized, while plants grown under LD continued to elongate; after 30 days, they were significantly taller (Fig. 1C). Similar to the main shoot, axillary branches developed under SD (branch numbers 15 and 20) were significantly shorter than equivalent branches that developed under LD (Fig. 1D–F).

For a better understanding of the structural differences between plants grown under LD and SD, we recorded the number of distinguishable nodes and internode lengths. During the first 3 weeks of cultivation, the number of nodes on plants grown under LD and SD was similar. However, the number of distinguishable nodes in cannabis plants grown under LD increased significantly relative to plants grown under SD, which produced condensed inflorescences (Fig. S1A). Furthermore, differences in internode length on the main stem were noted (Fig. S1B). Internode number 6, which developed before the transition to SD, had a similar length under LD and SD conditions (Fig. S1B and C). In contrast, significant differences were recorded in internode number 9 (Fig. S1B and D), which was the youngest internode on the day of transition from LD to SD, and internode number 12, which underwent full development under SD conditions (Fig. S1B and E). After 30 days, both internodes 9 and 12 were longer in plants grown under SD, whereas internode 18 was much longer in plants grown under LD (Fig. S1C–F). These results indicate that the photoperiod regime has a strong effect not only on inflorescence initiation, but also on node elongation and plant architecture. SD led to the rapid elongation of internodes 9 and 12, which were young at the time of the photoperiod transition, but to a strong reduction in elongation of newly formed internodes, which developed under SD (internode 18).

### Impact of photoperiod shifts on cannabis reproductive commitment and inflorescence architecture

**Figure 2:**
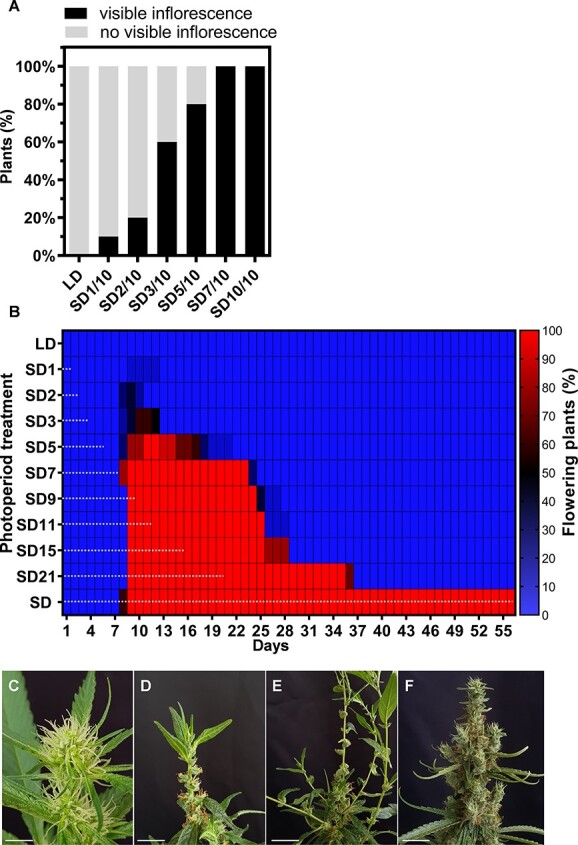
Development and commitment of cannabis plant inflorescences exposed to different photoperiod regimes. **A,** Percentage of flowering plants after 10 days under different combinations of long-day and short-day photoperiods (LD and SD, respectively). Numbers next to ‘SD’ indicate number of days under SD before plants were transferred to LD. Visible inflorescence is defined by the identification of three pairs of visible stigmas (*n* = 10). **B,** Percentage of plants showing development of an inflorescence during 56 days under LD, SD, or alternating photoperiods. Numbers following SD and horizontal dashed black lines indicate number of days under SD conditions before being transferred to LD. Color bar indicates percentage of plants with a developing inflorescence. Visible inflorescence was defined by the identification of three pairs of visible stigmas (*n* = 10). **C–E,** Representative inflorescence of an individual cannabis plant under **(C)** SD15 treatment (exposure to 15 days of SD), **(D)** on Day 30 (SD15 + LD15), and **(E)** on Day 50 (SD15 + LD35)**. F,** Inflorescence of control plant grown continuously under SD. Bar = 2 cm.

To identify the minimal number of days under SD required for the photoperiodic signal to be perceived and processed, plants grown under LD for 3 weeks were exposed to 1–7 days of SD and then transferred back to LD to complete 10 days of growth. The control plants were grown continuously under LD or SD for 10 days (Table S1). We found that 1 day under SD (SD1/10) was sufficient to stimulate inflorescence initiation in 10% of the plants (Fig. 2A). Extended exposure to SD resulted in a higher number of plants with inflorescences. Thus 60%, 80%, and 100% of the plants exposed to 3, 5, and 7 days of SD developed an inflorescence on day 10, respectively. As expected, plants exposed only to LD did not produce inflorescences (Fig. 2A). Because inflorescences had initiated in >50% of the plants after 3 days of SD, we defined this as the minimal SD required for inflorescence initiation.

The inflorescence branching pattern primarily depends on developmental signals within inflorescence meristems and their derived meristems. Each meristem undergoes a developmental choice between maintaining indeterminacy and committing to the floral fate. To deepen our understanding of how photoperiod affects cannabis inflorescence development, particularly how varying durations of short-day (SD) exposure followed by a return to long-day (LD) conditions influence reproductive commitment and inflorescence development, plants were exposed to 1–21 days of SD (SD1–SD21) followed by a transition back to LD over a total growth period of 56 days (Table S1). In plants exposed to 1–3 days of SD, an inflorescence was identified after 8–9 days, but its development was arrested after the transition to LD, and reverted to the LD growth pattern (Fig. 2B–F; Fig. S2). In plants exposed to 5–21 days of SD (SD5–SD21), visible inflorescences were initiated on all plants but did not persist after the transition to LD conditions, and the LD growth pattern was restored (Fig. 2; Fig. S2). After the transition to LD, the condensed inflorescences that had developed under SD disassembled into solitary flowers as a result of internode elongation (Fig. S2). For example, the inflorescences of SD15 plants were fully developed on the day of transition back to LD. However, after 13 days under LD (Day 28), inflorescence development ceased, and simple leaves developed at the top of the inflorescence in parallel with internode elongation, separating the condensed clusters of flowers into solitary flowers (Fig. 2C–F; Fig. S2B). Further growth was characterized by an LD growth pattern with elongated internodes (Fig. S2B – Week 7 for the SD15 group). Similar growth patterns were observed in plants that were grown under SD for prolonged periods. For example, plants that were grown under SD for 37 days (SD37) or 44 days (SD44), followed by LD, exhibited new leaf growth on the top of the main inflorescence of SD37 plants by Day 56 (Fig. S3A). However, at the same time, no such growth was observed on SD44 inflorescences or on control plants exposed only to SD (Fig. S3B and C). Eventually, both SD37 and SD44 plants that were left under LD for a longer time (until Day 72) showed LD growth patterns, with new shoots emerging from the inflorescences of SD44 plants (Fig. S3D–G). The strong effect of the transition back to LD on plant architecture and inflorescence development implies that the commitment to inflorescence development does not persist, and a continuous SD regime is required to inhibit internode elongation and maintain a condensed inflorescence structure.

### GA and auxin levels in the shoot apex are regulated by photoperiod

**Figure 3:**
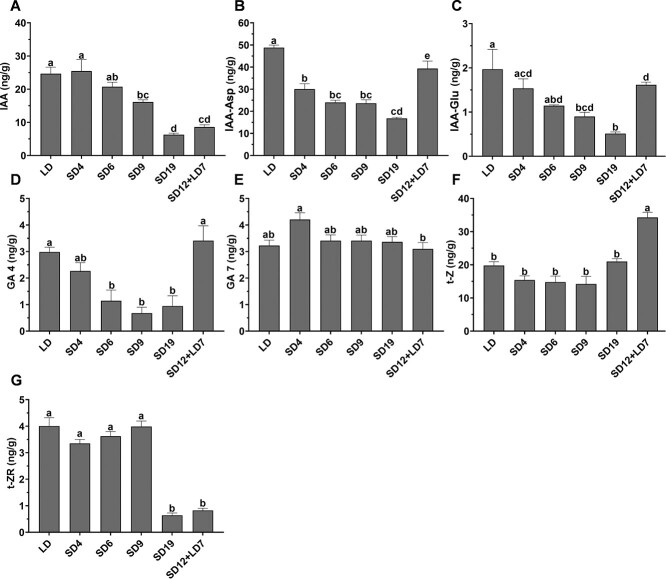
Levels of auxins, gibberellins (GA), and cytokinins in the shoot apex of cannabis plants under different photoperiod regimes. Levels of auxin derivatives: (**A**) IAA, (**B**) IAA-Asp, (**C**) IAA-Glu. Levels of gibberellins: (**D**) GA_4_, (**E**) GA_7_. Levels of cytokinins: (**F**) t-Z, (**G**) t-ZR. **H,** Levels of abscisic acid (ABA). Significant differences were assessed by one-way ANOVA, Tukey’s multiple comparison test, and are indicated at *P* ≤ 0.05. Presented data are averages ± SE (*n* = 4).

To evaluate the effect of photoperiod on the levels of endogenous phytohormones in cannabis, we analyzed the levels of auxin, GA, cytokinin, and their derivatives in the shoot apex of plants exposed to 4, 6, 9, or 19 days of SD; or grown under 12 days of SD followed by 7 days of LD. Plants cultivated under continuous LD were used as controls (Table S1). In plants exposed to SD conditions, gradual decreases in the levels of indole-3-acetic acid (IAA), IAA-aspartate (IAA-Asp), IAA-glutamate (IAA-Glu), and GA_4_ were recorded (Fig. 3 A–D). However, in plants exposed to 12 days of SD and then returned to LD (SD12 + LD7), levels of IAA-Asp, IAA-Glu, and GA_4_ were much higher than in plants exposed to 19 days of SD (Fig. 3 B–D). IAA level also increased, but to a much lower extent (Fig. 3A). The level of GA_7_ did not change markedly between the different treatments (Fig. 3E). Significant changes were found in cytokinin levels, but with no clear trend, most likely as a secondary effect of alterations in the levels of IAA and/or GA_4_ (Fig. 3 F–G). These results demonstrate that the levels of auxin and GA_4_ are regulated by photoperiod in the cannabis shoot apex. The reversible effect of LD on their levels further establishes the requirement of continuous SD for inflorescence development, as was also demonstrated by the morphological analysis (Fig. 2).

### Effect of exogenous plant growth regulators on plant morphology and cannabinoid content

**Figure 4:**
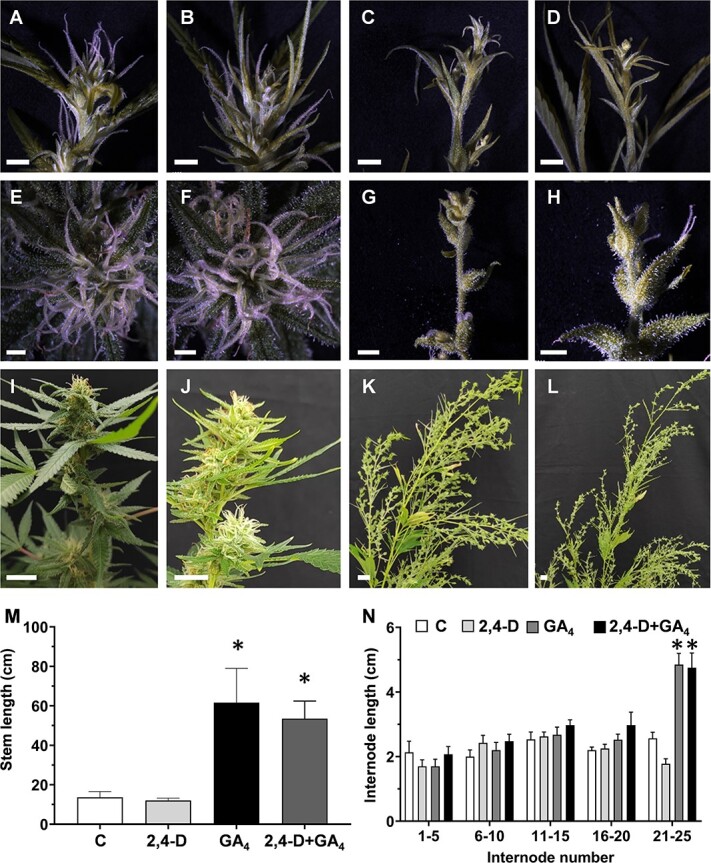
Effect of exogenous hormones on plant and inflorescence architecture under short-day photoperiod. Shoot apex of cannabis plants 13 **(A–D),** 28 **(E–H),** and 33 **(I–L)** days after plant growth regulator application. **A, E, I,** Control, (**B, F, J**) 4.5 ppm of synthetic auxin 2,4-D, (**C, G, K**) 100 ppm gibberellic acid GA_4_, (**D, H, L**) 100 ppm GA_4_ + 4.5 ppm 2,4-D. Bar = 2 mm for (**A–H**), and 2 cm for (**I–L**). **M,** Stem length and (**N**) internode length 42 days after hormone application. Significant differences between each treatment and the control (C) were determined by one-way ANOVA, Dunnet’s test, ^*^P ≤ 0.05. Presented data are averages ± SE (*n* = 3–4 for **M**; *n* = 15–20 for **N**).

To evaluate the role of plant growth regulators (PGRs) in inflorescence development, we applied the synthetic auxin 2,4-dichlorophenoxyacetic acid (2,4-D) and GA_3_ at the time of transition from the LD to SD. Application of GA_3_ or auxin+GA_3_ led to a delay in inflorescence development under SD. Thus, after 13 days of SD, developed inflorescences were observed in control and auxin-treated plants, while only solitary flowers were identified in GA_3_- and auxin+GA_3_-treated plants (Fig. 4A–D). After additional time under SD, compact inflorescences developed in control (Fig. 4E, I; Fig. S4A) and auxin-treated plants (Fig. 4F, J; Fig. S4B), while elongated and stretched inflorescences developed in plants treated with GA3 (Fig. 4G, K; Fig. S4C) and auxin+GA3 (Fig. 4H, L; Fig. S4D). Remarkably, single-inflorescence branches of the plants treated with GA_3_ were most similar to the single-inflorescence branches that developed in plants exposed to alternate photoperiods (Fig. S5). As expected, plants treated with GA_3_ alone or in combination with 2,4-D showed significant differences in plant architecture, including longer stems and internodes than in control plants (Fig. 4M, N; Figs. S4 and S5). Hence, the reduced level of GA under SD is associated with a reduction in branch elongation and the typical condensed inflorescence. The involvement of auxin in inflorescence development was not established and future studies are required to further evaluate its role in this process.

**Figure 5:**
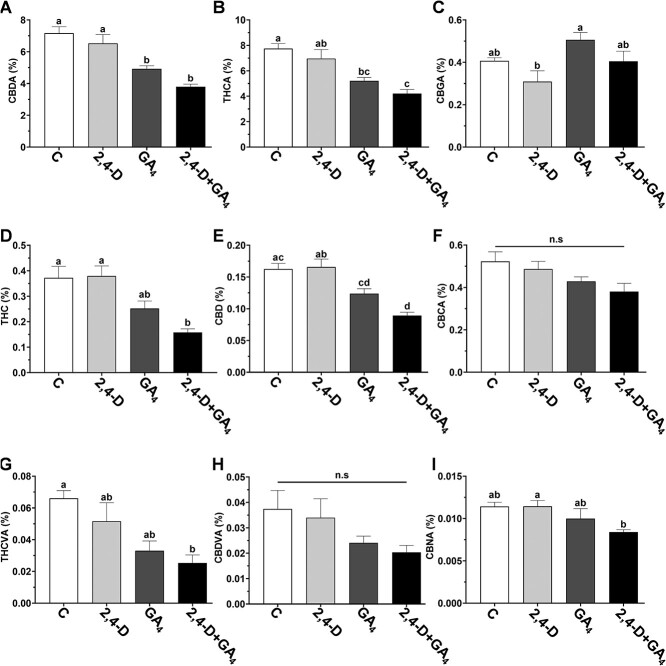
Effect of exogenous hormones on the concentration of major cannabinoids in cannabis plants grown under short-day photoperiod. Cannabinoids were analyzed in 10-cm apical sections of main stem inflorescences. **A,** CBDA, (**B**) THCA, (**C**) CBGA, (**D**) THC, (**E**) CBD, (**F**) CBCA, (**G**) THCVA, (**H**) CBDVA, (**I**) CBNA. Different letters above the bars represent significant differences between treatments by one-way ANOVA, Tukey’s multiple comparisons test, *P* ≤ 0.05; ns: not significant. Presented data are averages ± SE (*n* = 3–4). Cannabinoid concentrations were expressed as a percentage of the inflorescence dry weight.

To evaluate the involvement of GA and auxin in cannabinoid metabolism, their levels were measured in the inflorescences of treated plants (Fig. 5). Because inflorescence structure varied dramatically between treatments, only solitary flowers and bracts separated from the stems were sampled for the HPLC analysis. Levels of CBDA, THCA, THC, and CBD decreased in inflorescences of plants treated with GA_3_ and auxin+GA_3_ compared to control plants (Fig. 5A, B, D, E). However, THCVA level was only significantly lower in plants treated with auxin+GA_3_ compared to control plants (Fig. 5G). The levels of CBGA, CBCA, CBDVA, and CBNA were not significantly different from control plants (Fig. 5C, F, H, I). We conclude that the application of exogenous GA_3_ and auxin+GA_3_ negatively affects the cannabinoid content, suggesting that these phytohormones are involved in the regulation of cannabinoid metabolism in the inflorescences.

## Discussion

Photoperiod is a very powerful environmental factor in the plant’s annual cycle, controlling both vegetative and reproductive traits. It regulates the morphogenesis of these traits, e.g. flowering, tuberization, bulb formation, and hypocotyl elongation, as well as bolting of biannual plants [23–31]. For a long time, *C. sativa* L. was considered a short-day species that initiates inflorescence development in response to SD [32, 33]. We previously demonstrated that single-flower initiation and differentiation occur independently of photoperiod, but that photoperiod plays a fundamental role in orchestrating the events leading to inflorescence development [8]. However, the specific mechanisms underlying this regulation remained to be elucidated.

The current report provides new insights into the effect of SD on plant and inflorescence structure, hormonal balance, and cannabinoid accumulation. The exposure to SD affected cannabis plants in two phases. The first included rapid elongation of the internodes and main stem, from Day 5 to Day 10 of plant cultivation under SD (Fig. 1; Fig. S1). During the second phase, elongation of newly developed internodes ceased and a condensed inflorescence was formed (Fig. S1). The photoperiodic signal was perceived during the first 3 days under SD, but exposure to SD followed by LD reversed the process of inflorescence establishment and induced internode elongation within the inflorescence, reverting to the LD growth pattern, including the architecture of leaves and bracts, and the appearance of solitary flowers. At late stages of inflorescence maturation, LD induced the development of shoots that emerge from the mature inflorescence (Figs. S2 and S3). Similar shoots developed from dormant meristems subtending the cannabis inflorescence’s florets when cultured *in vitro* under LD [34]. These newly identified dormant meristems are most likely to have developed into the shoots emerging from the mature inflorescence under controlled-environment conditions as well (Figs. S2 and S3).

One of the most notable functions of auxin in plant growth is its regulation of apical dominance and shoot branching [35, 36], cell division and expansion, and stem elongation [20, 37, 38]. In the apex of cannabis exposed to SD, levels of IAA and its conjugates IAA-Asp and IAA-Glu, and of the active GA_4_ gradually decreased. These changes might be associated with the SD-induced morphological changes, including shorter internodes, intensive branching, reduction in petiole and leaf size, and alteration of the transition of compound to simple leaves development [8, 10, 12]. It has been established that gibberellin 2-oxidases that deactivate bioactive GAs or their precursors are regulated by photoperiod in dicots. In spinach, under SD, deactivation of GA by gibberellin 2-oxidases is needed for rosette development. Upon exposure to LD, active GA biosynthesis increases, resulting in stem elongation and flowering [39]. Similarly, in cannabis, the levels of GA_4_ decreased continuously under SD, but the transition back to LD resulted in full recovery of its levels (Fig. 3). This increase was also observed for auxin and its conjugates, further supporting the notion that the levels of these hormones in the shoot apex are regulated by photoperiod. These results explain the continuous requirement for SD conditions from inflorescence initiation to maturation (Fig. 2; Figs. S2 and S3). When we applied exogenous GA under SD, it acted similarly to GA accumulated in the shoot apex under LD. This led to the development of inflorescence architecture similar to that in plants shifted back to LD (Figs. 2 and 4; Figs. S2, S3, S4, and S5). In an *in vitro* assay in cannabis, GA also reduced flower development under SD in a dose-dependent manner, with plants grown on 10 μM GA_3_ failing to produce inflorescences [22].

Application of the GA-biosynthesis inhibitor paclobutrazol (PBZ) under SD induced higher density inflorescences and increased floral weight and harvest index of female inflorescences, both *in vivo* and *in vitro* [22, 40]. Similarly, in a study on *Camelina sativa*, PBZ application induced conspicuous changes in inflorescence morphology, which resulted in a compact raceme with a higher number of branches and flowers [41]. PBZ application to cannabis plants under LD did not induce inflorescence development (unpublished), further demonstrating the complex nature of inflorescence induction under LD, and may indicate the need for integration of other signals simultaneously and/or in specific regions of the shoot apex.

Application of exogenous GA also resulted in lower levels of cannabinoids accumulated in the inflorescence. This suppressive effect can be attributed to changes in glandular trichome properties, such as density or identity. While GA has been shown to positively regulate trichome development on vegetative organs in a number of species, its involvement in the development of different types of trichomes in cannabis inflorescences has yet to be determined [42]. A previous study in cannabis suggested that GA might play a direct inhibiting role on cannabinoid metabolism. Shortly after application of GA (72 h from first application) on flowering plants, the levels of THC decreased in leaves and flowers [43]. This suggests a direct suppressing role of GA on cannabinoid metabolism, similar to its negative role in the production of other specialized metabolites, such as volatile phenylpropanoids and carotenoids [14, 44]. The direct role of GA in cannabinoid metabolism is further supported by findings that GA application activates the cannabis promoter of *AROMATIC PRENYLTRANSFERASE 4* (*PT4*), which catalyzes CBGA biosynthesis, as demonstrated in transgenic *Arabidopsis* [45]. However, *PT4* promoter activation by GA may highlight the complexity of hormonal regulation on cannabinoid biosynthesis, potentially indicating short-term negative feedback loops or inherent differences in promoter activity between plant species. In contrast to the strong effect of the exogenous GA, application of 2,4-D did not result in significant changes in plant architecture, inflorescence development, or cannabinoid content (Figs. 4 and 5; Fig. S4). In a previous study, continuous application of 1-naphthaleneacetic acid (NAA) in cannabis also had no effect on cannabinoid levels [46]. However, contradictory results have been documented on the impact of different concentrations of NAA on cannabis plant development, including no effect, increases and decreases in plant height, and in the length of axillary branches [47, 48]. Different reactions to auxin treatment may depend on the specific auxin used, the duration of application, its concentration, the application method, and the environmental and physiological conditions and genetic background of the treated plants. Further experiments considering these factors are needed to elucidate its function in reproductive development in cannabis.

Gaining insight into the physiological and genetic regulatory networks that control inflorescence development in cannabis could yield more efficient production strategies. However, since our study focused on one commercial cultivar, it is worth considering the possibility that these findings may vary across different cannabis cultivars. Overall, perception of the photoperiodic signal by the cannabis plant requires at least 3 days of SD, and then SD is continuously required to maintain a low GA level at the shoot apex. This decrease in GA level reduces stem and internode elongation, which in turn allows solitary flowers to develop close to one another, resulting in a condensed inflorescence.

## Materials and methods

### Plant materials and growth conditions

Medical cannabis cultivar A was obtained from Canndoc LTD, a commercial medical cannabis cultivation company in Israel. Rooted cuttings were planted in 200-ml pots and grown in an environmentally controlled growing room at 24°C–26°C under LD (18/6 h light/dark). Then plants were transferred to 3-l pots, one plant per pot, in a 1:1 mixture of perlite 2-1-2 (Agrekal, Habonim, Israel) and Green 63 (Even Ari Green, Beit Elazari, Israel) under the same photoperiod. Plants were transferred to SD (12/12 h light/dark) as indicated for each specific experiment. The photoperiod regime for each experiment is detailed in Table S1. Light for the LD was supplied by metal halide bulbs (1000 W), with a light intensity of 600 μmol m^−2^ s^−1^ (Tru Blue, Growlite, Glendale, AZ, USA). Light for the SD was supplied by high-pressure sodium bulbs (1000 W) with a light intensity of 1000 μmol m^−2^ s^−1^ (Real Red HPS, Growlite, Glendale, AZ, USA). In the hormone analysis experiment HPS bulbs were used for plant cultivation in both LD and SD growth phases. Irrigation was supplied via 1 l h^−1^ discharge-regulated drippers (Plastro-Gvat, Kibbutz Gvat, Israel), 1 dripper per pot. The volume of irrigation was 500–800 ml pot^−1^ day^−1^, set to allow 35%–40% drainage. Fertilizers were supplied by fertigation, i.e. dissolved in the irrigation solution at each irrigation event at concentrations of 85 ppm N (with 1:2 ratio of NH_4_^+^:NO_3_^−^), 40 ppm P_2_O_5_ (17 ppm P), and 108 ppm K_2_O (90 ppm K). Micronutrients were supplied chelated with EDTA at 0.4 ppm Fe, 0.2 ppm Mn, and 0.06 ppm Zn. Cultivar A was used for the physiological measurements, requirements for inflorescence initiation, evaluation of reproductive meristem commitment, hormone analysis, and exogenous hormone treatments.

### Morphological and physiological parameters

Plant morphological measurements were conducted routinely every 4 days, throughout the experiments. Twenty plants were initially exposed to LD conditions for 7 days. Following this, they were divided into two groups: one group was maintained under continuous LD conditions, while the other group was subjected to SD conditions for a period of 30 days (Table S1). ‘Visible inflorescence’ was defined as the stage at which at least three pairs of stigmas became visible in the apical part of the shoot [8]. The presence of flowers at the shoot apex and inflorescence development were evaluated daily using an illuminated loupe (40×, 25 mm). Stem length was measured from the base of the plant to the top of the main stem. Internodes and branches were numbered sequentially, with the first node above the ground as 1. Internode length (bract to bract) was measured with a measuring tape.

### Cannabinoid analysis

For the analysis of cannabinoid concentrations in plants treated with exogenous phytohormones, the top inflorescence of the main stem was sampled from mature inflorescences 44 days after the beginning of SD conditions. Due to the significant variation in inflorescence structure between treatments, only solitary flowers and bracts separated from the stems were sampled. Following dissection from the plants, the inflorescences were immediately hand trimmed and dried in the dark at 19°C and 45% relative humidity for 17 days. The samples were cured for 2 months in sealed plastic bags that were mixed and opened once daily for 15 min as is customary in the cannabis industry. For cannabinoid analysis the dried inflorescences were extracted as is described by Danziger and Bernstein [49], and cannabinoid concentrations in the extracts were analyzed as is described by Saloner and Bernstein [50]. In short, the dried inflorescence samples were ground manually to a homogeneous mixture, and for each sample, 50 mg of the ground plant tissue was placed in a 50-ml centrifuge tube, with 10 ml ethanol ABS A.R. (Gadot-Group, Netanya, Israel). The tubes were shaken for 1 h at room temperature and then centrifuged for 15 min at 5000 rpm (Megafuge 16 centrifuge, Thermo-Scientific, Waltham, MA, USA). The resulting supernatant was filtered through a 0.22-μm PVD filter (Bar-Naor Ltd., Ramat Gan, Israel) and then analyzed by HPLC (Jasco 2000 Plus series HPLC system) to determine cannabinoid concentrations.

### Phytohormone analysis

The plants cultivated under LD for 3 weeks were randomly divided into six groups of 50 plants. Each group was exposed to different photoperiod regimes: SD for 4, 6, 9, and 19 days; or 12 days of SD followed by 7 days of LD; and continuous LD as a control. Four biological replicates were collected from each plant treatment group. Every replicate was a mix of apical meristems from 5 to 15 plants with a total weight of 200 mg. For each sample, shoot apex (10 mm) with removed leaves was collected, weighed, and immediately frozen in liquid nitrogen and stored at −80°C. Extraction of plant hormones and LC–MS–MS analyses were conducted according to Sood et al. [51] using a UPLC-Triple Quadrupole-MS (Waters Xevo TQ MS) and a Waters Acquity UPLC BEH C18 1.7 μm 2.1 × 100 mm column with a VanGuard precolumn (BEH C18 1.7 μm 2.1 × 5 mm).

### Exogenous application of PGRs

Plants were cultivated for 60 days under LD condition (as described in plant materials and growth conditions section). Then, the plants were randomly divided into four groups of four plants each. The groups were treated as follows: sprayed until run-off with the synthetic auxin 2,4-dichlorophenoxyacetic acid (2,4-D, Citrus Fix, AMVAC, Los Angeles, CA, USA) and 0.1% (v/v) of the surfactant BB5, or drenched with 250 ml of a 100 ppm GA_3_ (Giberllon, Gadot Agro, Kidron, Israel) solution, or treated with a mixture of both treatments. A control group was sprayed with distilled water containing 0.1% BB5. At the time of PGR application, the photoperiod was adjusted to SD conditions (Table S1). All samples were analyzed 44 days after application of PGRs.

### Statistical analyses

All statistical analyses were performed utilizing GraphPad-Prism 8.0.2 software. Specific statistical tests and the number of biological replicates used are described within the corresponding figure legends.

## Supplementary Material

Web_Material_uhae245

## Data Availability

All created data are presented in this article and in the supplementary materials.
