# Supplementary material for: Inflorescence development in female cannabis plants is mediated by photoperiod and gibberellin
Source: Hortic Res. 2024 Sep 3;11(11):uhae245. doi: 10.1093/hr/uhae245 (PMC11560369; doi:10.1093/hr/uhae245)
Supplement: Web_Material_uhae245 [file web_material_uhae245.zip › Sup Table and Figures.pdf]

Table S1. Experimental designs of photoperiodic treatments

Figures	Experiment	Name of Treatment	Photoperiod Phase 1	Photoperiod Phase 2
Figure 1; Figure S1	Development of cannabis plants under different photoperiods	LD	30 LDs	-
		SD	30 LDs	-
Figure 2A	commitment of cannabis plant inflorescences exposed to different photoperiod regimes.	LD	10 LDs	-
		SD1/10	1 SD	
		SD2/10	2 SDs	
		SD3/10	3 SDs	7 LDs
		SD5/10	5 SDs	5 LDs
		SD9/10	9 SDs	1 LD
		SD10/10	10 SDs	-
Figure 2B; Figure S2	Development and commitment of cannabis plant inflorescences exposed to different photoperiod regimes.	LD	56 LDs	-
		SD1	1 SD	55 LDs
		SD2	2 SDs	54 LDs
		SD3	3 SDs	53 LDs
		SD5	5 SDs	51 LDs
		SD7	7 SDs	49 LDs
		SD9	9 SDs	47 LDs
		SD11	11 SDs	45 LDs
		SD15	15 SDs	41 LDs
		SD21	21 SDs	35 LDs
		SD	56 SDs	-
Figure 3	Levels of auxins, gibberellins (GA) and cytokinins in the shoot apex of cannabis plants under different photoperiod regimes	LD	1 LDs	-
		SD4	19 SDs	-
		SD6	19 SDs	-
		SD9	19 SDs	-
		SD19	19 SDs	-
		SD12+LD7	12 SDs	7 LDs
Figures 4 and 5	Effect of exogenous hormones	All treatments	44 SDs	-
Figure S3	Development and commitment of inflorescences of cannabis plants exposed to long days following prolonged growth under short-day photoperiod.	SD37	37 SDs	35 LDs
		SD44	44 SDs	28 LDs
		SD	72 SDs	-
Figure S4	Effect of exogenous hormones on plant architecture under short-day photoperiod	All treatments	33 SDs	-

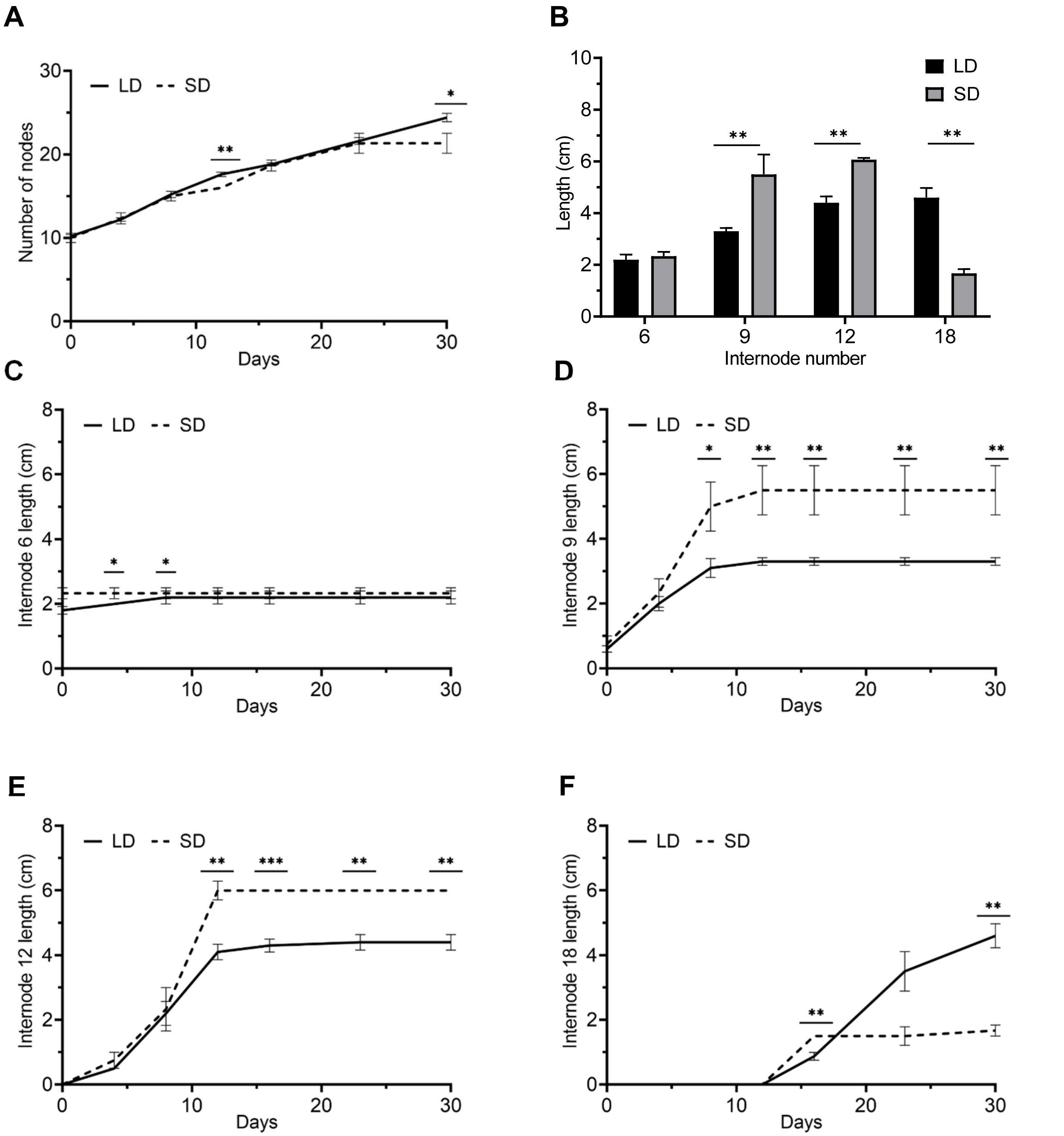

Figure S1. Number of nodes and internodes in cannabis plants grown under long-day and short-day photoperiods (LD and SD, respectively). **A** Number of nodes during 30 days of growth under LD and SD. **B** Internode lengths after 30 days of growth under LD and SD (internode numbers: 6, 9, 12, 18). Plants that were transferred to SD had 9 internodes on the day of their transfer. **C–F** Internode elongation during 30 days of growth under LD and SD (internode numbers: 6, 9, 12, 18, respectively). Significant differences were assessed by unpaired t-test; *, **, ***, $P \leq 0.05$, 0.01 and 0.001, respectively. Presented data are averages ± SE (n = 3–5).

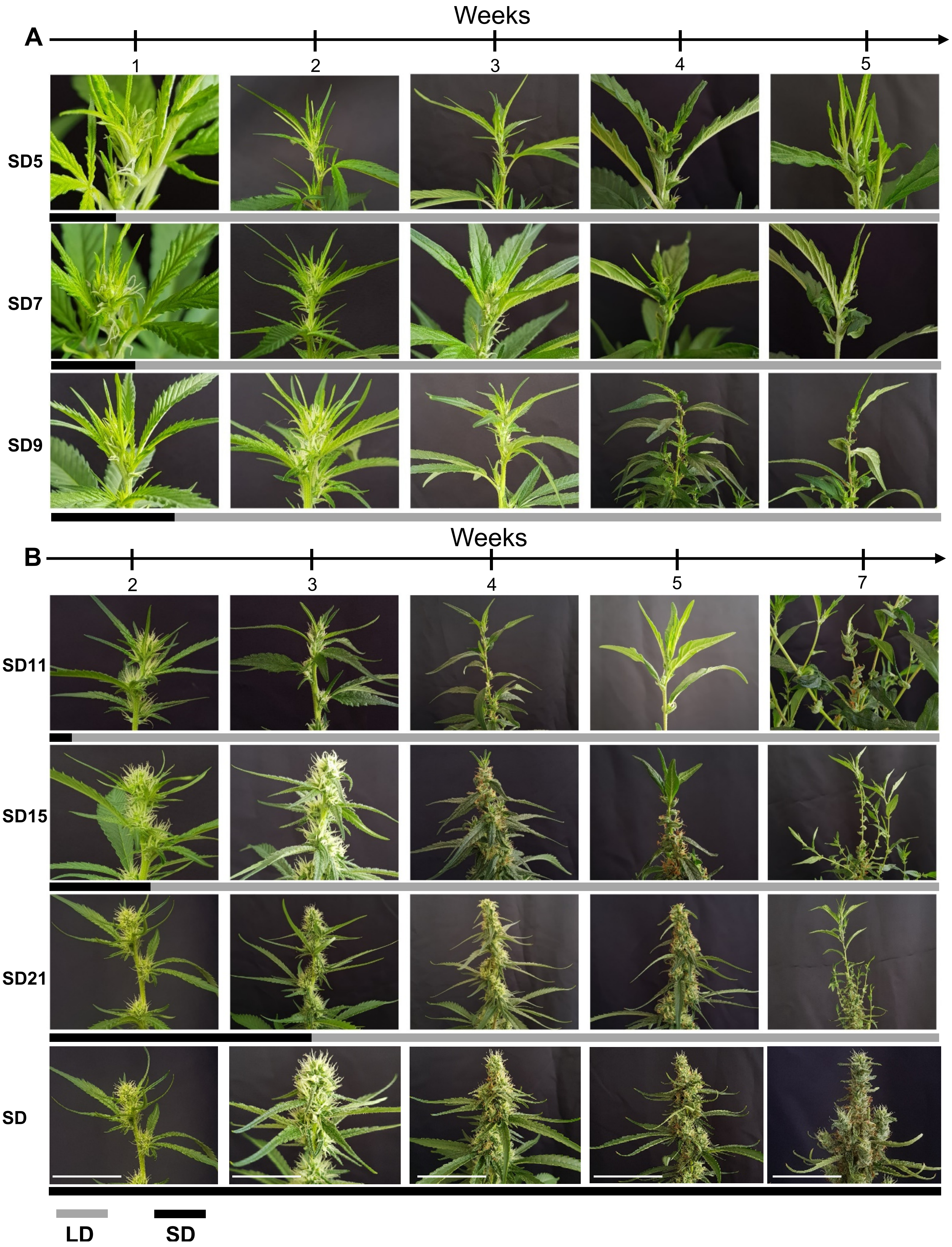

Figure S2. Development and commitment of inflorescences in cannabis plants exposed to different photoperiod regimes. Representative images of individual shoot apexes of plants exposed to continuous short-day photoperiod (SD) or alternating photoperiods. The numbers following "SD" and the horizontal black lines indicate the number of days under SD before being transferred to long-day photoperiod LD (gray line). The time of imaging in weeks from the beginning of the experiment is presented at the top of each panel. **A** SD5, SD7 and SD9. **B** SD11, SD15, SD21 and continuous SD as a control.

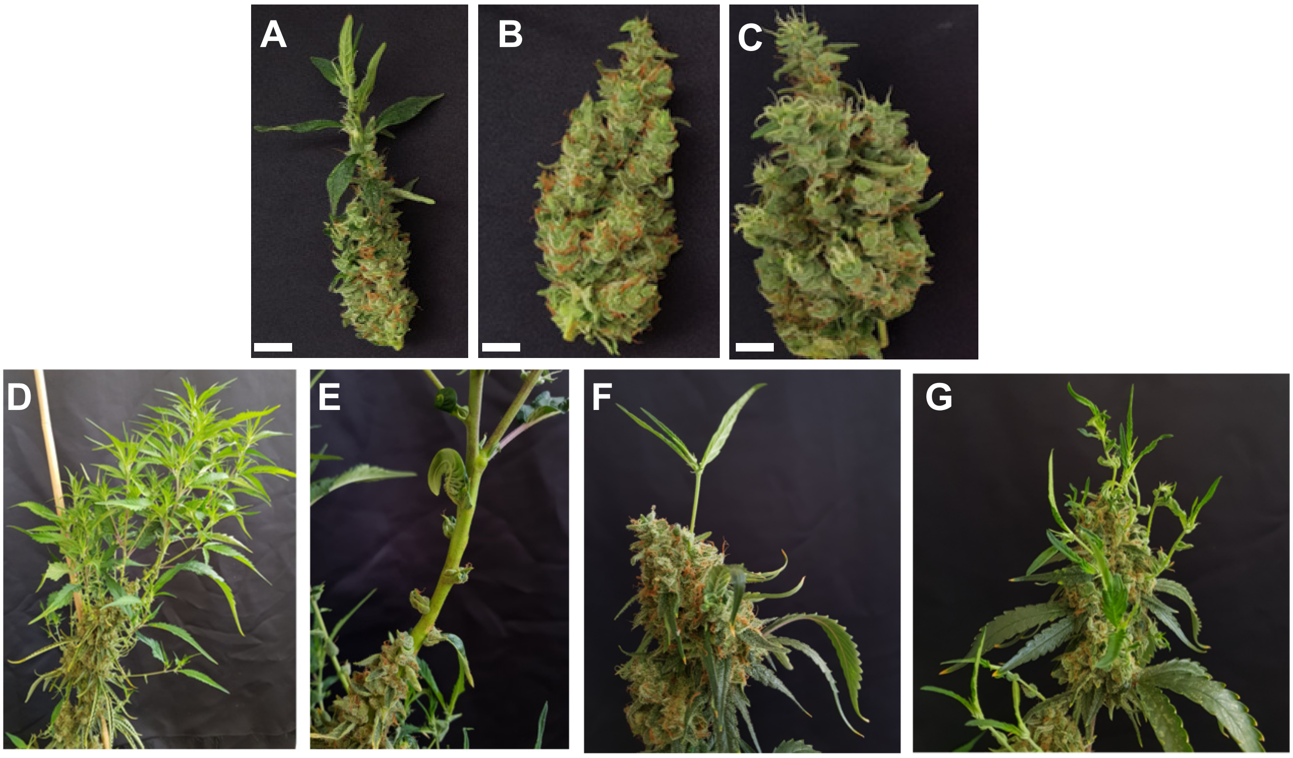

Figure S3. Development and commitment of inflorescences of cannabis plants exposed to long-day photoperiod (LD) following prolonged growth under short-day photoperiod (SD). Representative inflorescence of SD37 **(A, D, E)**, SD44 **(B, F, G)** and SD control plants **(C)** on day 56 **(A‑C)** and on day 72 **(D-G)**. Plants were grown under SD for 37 and 44 days followed by 19 **(A)**, 12 **(B)**, 35 **(D,E)** and 28 days under LD. **(D)** and **(E)** are the same individual. **F, G** Two individual plants. Bar = 1 cm.

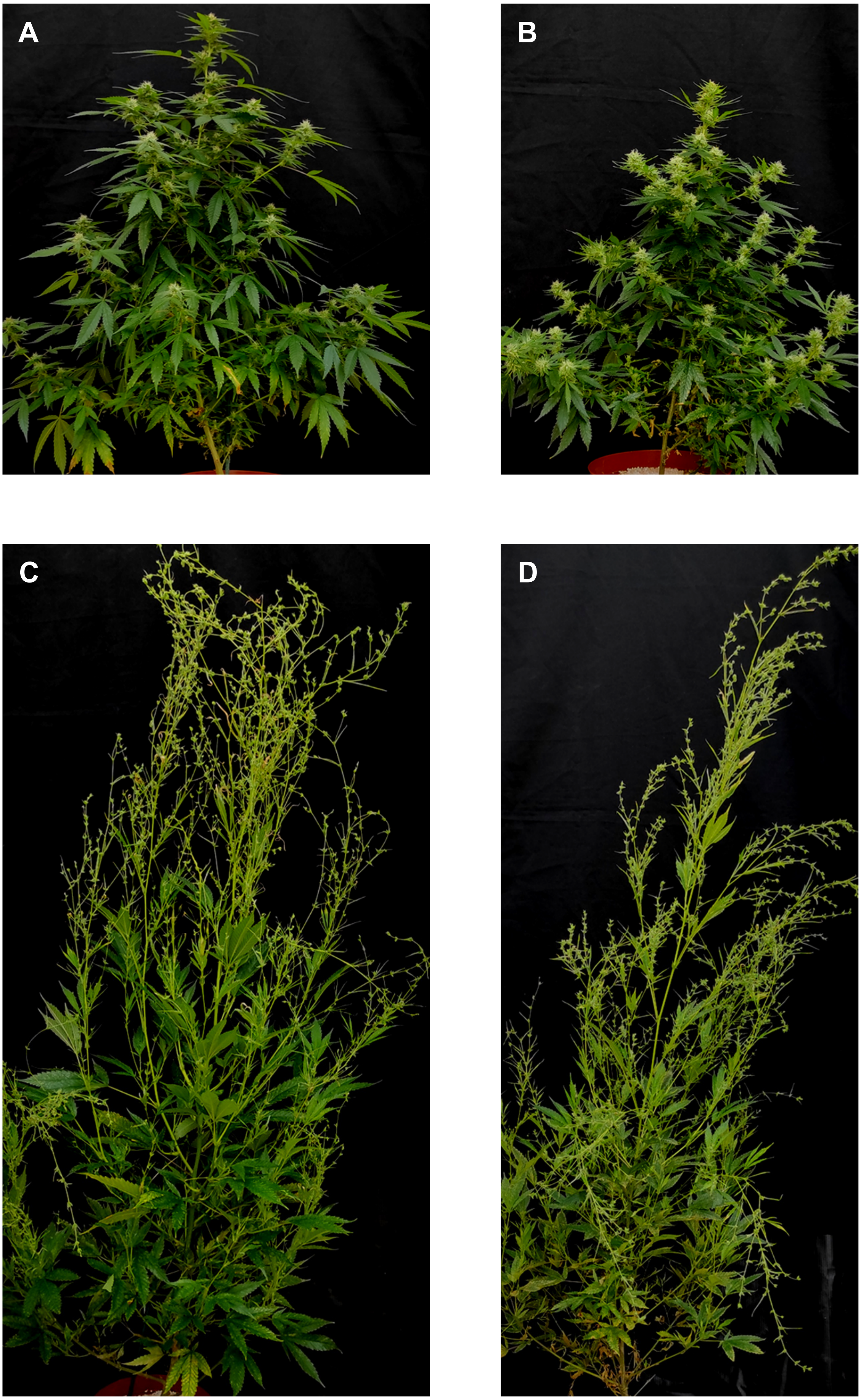

Figure S4. Effect of exogenous hormones on plant architecture under short-day photoperiod (SD). Cannabis plants grown under SD, 33 days after plant growth regulator application. **A** Control, **B** 4.5 ppm of synthetic auxin 2,4-D, **C** 100 ppm of gibberellin GA_4, **D** 100 ppm GA_4 + 4.5 ppm 2,4-D.

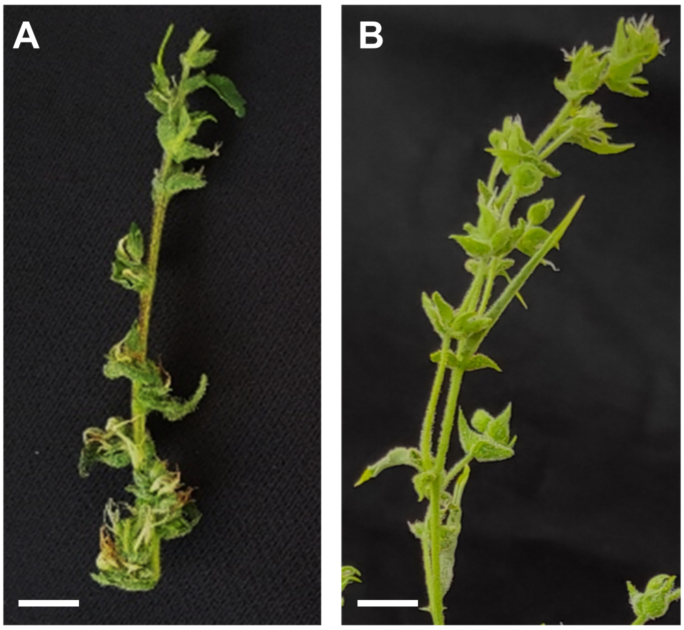

Figure S5. Comparison of shoot apexes of inflorescences of cannabis grown under alternating photoperiods and those treated with gibberellin. **A** Shoot apex of cannabis grown under 15 days of short-day photoperiod (SD) + 21 days of long-day photoperiod. **B** Shoot apex of cannabis 33 days after gibberellin application, under SD. Bar = 1 cm.
